# Management of Complicated Appendicitis During Pregnancy in the US

**DOI:** 10.1001/jamanetworkopen.2022.7555

**Published:** 2022-04-15

**Authors:** Matthew Ashbrook, Vincent Cheng, Kulmeet Sandhu, Koji Matsuo, Morgan Schellenberg, Kenji Inaba, Kazuhide Matsushima

**Affiliations:** 1 Division of Acute Care Surgery, Department of Surgery, University of Southern California, Los Angeles; 2 Department of Surgery, Cedars-Sinai Medical Center, Los Angeles, California; 3 Department of Obstetrics and Gynecology, University of Southern California, Los Angeles

## Abstract

**Importance:**

Data are sparse regarding the optimal treatment for complicated appendicitis during pregnancy.

**Objective:**

To compare nonoperative and operative management in complicated appendicitis during pregnancy.

**Design, Setting, and Participants:**

This cohort study was conducted using National Inpatient Sample data from between January 2003 and September 2015. This database approximates a 20% stratified sample of US inpatient hospital discharges. Included individuals were pregnant women discharged with the diagnosis of complicated appendicitis. Data were analyzed from February 2020 through February 2022.

**Exposures:**

Study patients were categorized into 3 groups: those with successful nonoperative management, failed nonoperative management with delayed operation, or immediate operation for complicated appendicitis.

**Main Outcomes and Measures:**

Clinical outcomes, including maternal infectious complications and perinatal complications, hospital length of stay, and total hospital charges.

**Results:**

Among 8087 pregnant women with complicated appendicitis (median [IQR] age, 27 [22-32] years), nonoperative management of complicated appendicitis was successful among 954 patients (11.8%) and failed among 2646 patients (32.7%), who underwent delayed operation; 4487 patients (55.5%) underwent immediate operation. In multivariate analysis, successful nonoperative management was associated with higher odds of amniotic infection (odds ratio [OR], 4.35; 95% CI, 2.22-8.53; *P* < .001) and sepsis (OR, 1.52; 95% CI, 1.10-2.11; *P* = .01) compared with immediate operation, while there was no significant difference in preterm delivery, preterm labor, or abortion. However, failed nonoperative management that required delayed operation was associated with higher odds of preterm delivery, preterm labor, or abortion compared with immediate operation (OR, 1.45; 95% CI, 1.24-1.68; *P* < .001). Immediate operation was associated with decreased hospital charges compared with nonoperative management that was successful (regression coefficient [RC], 0.09; 95% CI, 0.07-0.11; *P* < .001) and that failed (RC, 0.12; 95% CI: 0.11-0.14; *P* < .001). In subgroup multivariate logistic regression analysis, each day in delay to surgery was associated with an increase in odds of preterm delivery, preterm labor, or abortion by 23% (OR, 1.23; 95% CI, 1.18-1.29; *P* < .001).

**Conclusions and Relevance:**

This study found that immediate operation for complicated appendicitis in pregnant women was associated with lower odds of maternal infectious complications without higher odds of perinatal or other maternal complications compared with successful nonoperative management. Failed nonoperative management was associated with worse clinical outcomes.

## Key Points

**Question:**

What management strategy for complicated appendicitis among pregnant women is associated with decreased maternal infectious complications and perinatal complications?

**Findings:**

In this cohort study of 8087 pregnant women with complicated appendicitis, immediate operation was associated with lower odds of infectious complications, including amniotic infection and sepsis, compared with successful and unsuccessful nonoperative management. When nonoperative management failed and required delayed operation, it was associated with significantly higher odds of preterm labor, preterm delivery, or abortion.

**Meaning:**

These findings suggest that immediate operation may be the preferred management strategy for complicated appendicitis among pregnant women.

## Teaser

This cohort study investigates the associations of immediate operation and successful and failed nonoperative management for acute appendicitis with maternal and perinatal complications among pregnant women.

## Introduction

Acute appendicitis is one of the most common nonobstetrical emergencies in pregnant women. Acute appendicitis complication occurs in 1 in 700 to 1500 live birth pregnancies at some time during pregnancy, with most instances among women aged younger than 30 years.^1,2,3^ By the same token, more than 1 in 20 women of childbearing age who present with acute appendicitis are pregnant.^4^ Previous studies have found that appendectomy during pregnancy for uncomplicated acute appendicitis is safe.^5,6,7^ In fact, laparoscopic appendectomy for uncomplicated appendicitis has been associated with decreased maternal and fetal morbidity and has therefore become the recommended management strategy vs nonoperative management.^8,9,10,11^

Unlike uncomplicated appendicitis, complicated appendicitis (eg, perforation with peritonitis or abscess) among pregnant women does not have a clear optimal management strategy.^12,13^ For complicated appendicitis within the general population, immediate operation and nonoperative management, including antibiotics and percutaneous drainage for appendiceal abscess, have been found to be associated with effective outcomes.^14,15^ However, when nonoperative management failed, the need for open surgery and bowel resection portending to increased morbidity was increased.^16^ Current guidelines for the management of complicated appendicitis in the general population recommend laparoscopic appendectomy given that data suggest the feasibility and safety of laparoscopic appendectomy, with shorter hospital stays and decreased morbidity and mortality compared with nonoperative intervention.^6,17,18^ Given the scarcity of literature comparing management strategies for complicated appendicitis during pregnancy, the aim of this study was to compare clinical outcomes between nonoperative and operative management of complicated appendicitis in pregnant women. We hypothesized that immediate operation among pregnant women who presented with complicated appendicitis would be associated with decreased morbidity and hospital length of stay (LOS).

## Methods

This cohort study was approved and a waiver of informed consent granted owing to the use of deidentified data by the institutional review board at the University of Southern California. The Strengthening the Reporting of Observational Studies in Epidemiology (STROBE) reporting guideline was followed to summarize the performance of the observational study.

### Data Source and Patient Eligibility

This is a retrospective cohort study using Healthcare Utilization Project National Inpatient Sample (NIS) data from between January 2003 and September 2015. The NIS database is deidentified and approximates a 20% stratified sample of US inpatient hospital discharges.^19^ We queried the database for pregnant women with complicated appendicitis during the study period using diagnostic codes from the *International Classification of Diseases, Ninth Revision, Clinical Modification* (*ICD-9-CM*). Complicated appendicitis included *ICD-9-CM* codes 540.0 (acute appendicitis with generalized peritonitis) and 540.1 (acute appendicitis with peritoneal abscess) (Table 1).^20,21^ The study end date of September 2015 was chosen because *ICD-9-CM* was retired at this time in favor of the *International Statistical Classification of Diseases, Tenth Revision, Clinical Modification *(*ICD-10-CM*). Pregnant patients were identified using the neonatal and/or maternal (NEOMAT) variable built into the NIS database, which identifies patients who are discharged with neonatal and maternal diagnoses.^22^ A NEOMAT code of 1 includes only patients discharged with maternal codes and excludes neonatal codes. Patients who had pregnancies with complications, such as ectopic pregnancy and hydatidiform mole, were excluded using associated *ICD-9-CM* diagnosis codes (Table 1).

**Table 1.** Diagnosis and Procedure Codes

Diagnosis or procedure	*ICD-9-CM *codes
Appendicitis with generalized peritonitis	540.0
Appendicitis with peritoneal abscess	540.1
Hydatidiform mole	630
Ectopic pregnancy	633
Other abnormal pregnancy	631
Laparoscopic appendectomy	47.01
Other (open) appendectomy	47.09
Preterm labor, preterm delivery, or abortion	632, 634.00, 634.01, 634.02, 634.10, 634.11, 634.12, 634.20, 634.21, 634.22, 634.30, 634.31, 634.32, 634.40, 634.41, 634.42, 634.50, 634.51, 634.52, 634.60, 634.61, 634.62, 634.70, 634.71, 634.72, 634.80, 634.81, 634.82, 634.90, 634.91, 634.92, 644.0, 644.03, 644.10, 644.13, 644.20, 644.21, 656.4, V27.1
Antepartum hemorrhage	641.0, 641.01, 641.03, 641.1, 641.11, 641.13, 641.20, 641.21, 641.23, 641.30, 641.31, 641.33, 641.80, 641.81, 641.83, 641.90, 641.91, 641.93
Premature rupture of membranes	658.10, 658.11, 658.13
Infection of amniotic cavity	658.40, 658.41, 658.43
SIRS, sepsis, or severe sepsis	995.90, 995.91, 995.92
Pneumonia	480x-482x, 485-487x

Abbreviations: *ICD-9-CM*, *International Classification of Diseases, Ninth Revision, Clinical Modification*; SIRS, systemic inflammatory response syndrome.

### Baseline Demographic and Outcome Variables

Baseline patient and hospital characteristics were abstracted from NIS. These included age, hospital location, hospital region, race and ethnicity, payer, income quartile, and discharge year. Race and ethnicity categories reflect options provided for Agency for Healthcare Research and Quality Healthcare Cost and Utilization Project (HCUP) coding of data elements, which include Asian or Pacific Islander, Black, Hispanic, Native American, White, and other. The source of race and ethnicity classification was the HCUP State Inpatient Databases disparities analysis file, which is collected by self-report on admission to the hospital using fixed categories. Race and ethnicity were assessed in this study to investigate the association between race and ethnicity and management of complicated appendicitis in pregnant patients. Using coding algorithms validated for defining comorbidities in *ICD-9-CM* administrative data,^23^ a Charlson Comorbidity Index (CCI) score was identified for each patient. Clinical outcome variables included maternal death, preterm delivery, preterm labor, abortion, antepartum hemorrhage, premature rupture of membranes, amniotic infection, systemic inflammatory response syndrome, sepsis, severe sepsis, and pneumonia and reflect outcomes that occurred at any point during hospitalization (Table 1). Owing to the inability of *ICD-9-CM* codes to stratify patients by trimester, a composite perinatal outcome of preterm labor or delivery and abortion was used given that these perinatal outcomes are likely to be distributed differently across trimesters. Other outcome variables included hospital LOS and total hospital charges. The latter outcome was adjusted for inflation using the Consumer Price Index measured by the US Bureau of Labor Statistics.^24^

### Statistical Analysis

Using *ICD-9-CM* procedure codes, we divided study patients into 3 groups: those with successful nonoperative management, failed nonoperative management, and immediate operation for complicated appendicitis. Successful nonoperative management was defined as no appendectomy during the hospital stay. Failed nonoperative management was defined as a trial of at least 1 day of nonoperative management followed by operative intervention (ie, laparoscopic or open appendectomy). Patients with unknown operation timing were excluded from the study. Patient baseline characteristics were compared across groups using univariate analysis. The χ^2^ test of independence was used to examine associations between treatment strategy and dichotomous outcome variables. The Kruskal-Wallis test was used to examine associations between treatment strategy and continuous outcome variables.

Multivariate regression analysis was used to compare clinical and economic outcomes across groups after adjusting for baseline characteristics, including patient characteristics (ie, age, CCI score, and race and ethnicity), economic characteristics (ie, payer and income quartile), and hospital characteristics (ie, region, location, and teaching status). Multivariate logistic regression analysis was used to examine the association between treatment strategy and dichotomous outcome variables. Effect size was expressed with an adjusted odds ratio (OR) and corresponding 95% CI. Multivariate linear regression analysis was used for continuous outcome variables. To satisfy statistical assumptions necessary for linear regression, base 10 logarithmic transformations were required for hospital LOS and total charges.

Subgroup analysis was then performed on patients who underwent operative intervention. Multivariate regression analyses were performed to examine the association of delay in performing operation with maternal and perinatal outcomes after adjusting for the baseline characteristics previously listed. Effect size was expressed with an adjusted OR and corresponding 95% CI. Delay was defined as the number of days between hospital admission and operative intervention.

Weighted values for national estimates provided by NIS were used per the program’s recommendation. The complete case analysis (CCA) method was used for handling missing data; this has been shown to be an effective method for controlling relative bias when performing analysis of the NIS database.^25^ Statistical interpretation was based on a 2-tailed hypothesis, with a *P* value < .05 considered to be statistically significant. Per the HCUP data use agreement, analyses including 10 or fewer observations were suppressed. SPSS statistical software version 27.0 (IBM) and R statistical software version 3.5.3 (R Project for Statistical Computing) were used for all analyses. Data were analyzed from February 2020 through February 2022.

## Results

A total of 8087 pregnant women with complicated appendicitis (median [IQR] age, 27 [22-32] years; 225 [2.8%] Asian or Pacific Islander individuals, 644 [8.0%] Black individuals, 1986 [24.6%] Hispanic individuals, 81 [1.0%] Native American individuals, 3866 [47.8%] White individuals, and 394 [4.9%] individuals with other race or ethnicity) met our inclusion criteria (Figure). Nonoperative management of complicated appendicitis was successful among 954 patients (11.8%) and failed among 2646 patients (32.7%), who underwent delayed operation, representing 73.5% of the total 3600 women with nonoperative treatment; 4487 patients (55.5%) underwent immediate operation (Table 2). Immediate operation included 2345 patients (52.3%) who underwent laparoscopic appendectomy and 2142 patients (47.7%) who underwent open appendectomy, while failed nonoperative management of acute appendicitis included 1014 patients (38.3%) who underwent laparoscopic appendectomy and 1632 patients (61.7%) who underwent open appendectomy.

**Figure.**
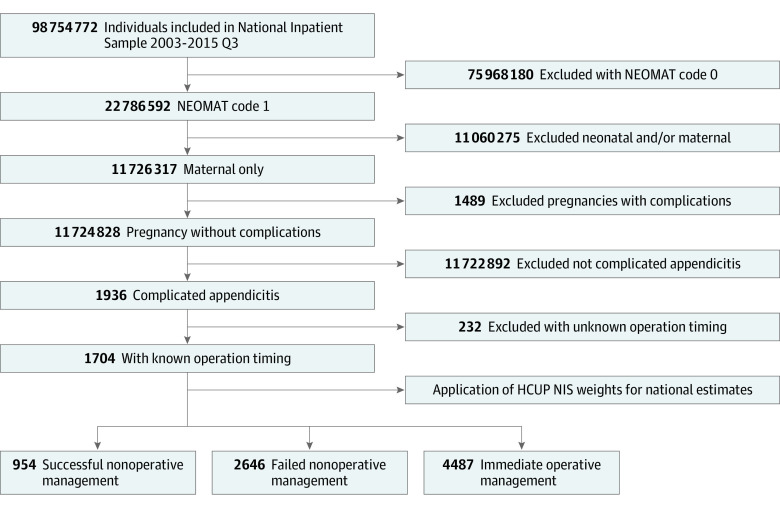
Patient Flowchart HCUP indicates Healthcare Cost and Utilization Project; NEOMAT, neonatal and/or maternal variable; NIS, National Inpatient Sample; Q3, quarter 3.

**Table 2.** Patient and Institutional Characteristics

Characteristic	Patients, No. (%)^a^			*P* value
	Immediate operation (n = 4487)	Successful NOM (n = 954)	Failed NOM (n = 2646)	
Age, median (IQR), y	27 (22-32)	29 (24-33)	27 (22-32)	<.001
CCI score ≥2	25 (0.6)	NA^b^	NA^b^	.03
Location				
Rural	355 (7.9)	48 (5.0)	211 (8.0)	<.001
Urban nonteaching hospital	1685 (37.5)	307 (32.1)	1078 (40.7)	
Urban teaching hospital	2418 (53.8)	594 (62.2)	1353 (51.1)	
Region				
Northeast	639 (14.2)	214 (22.4)	496 (18.7)	<.001
Midwest	908 (20.2)	243 (25.5)	390 (14.7)	
South	1757 (39.1)	342 (35.8)	1157 (43.7)	
West	1188 (26.4)	155 (16.2)	603 (22.8)	
Race and ethnicity^c^				
Asian or Pacific Islander	157 (3.9)	15 (1.9)	53 (2.3)	<.001
Black	292 (7.2)	131 (16.7)	221 (9.4)	
Hispanic	1095 (27.0)	189 (24.0)	702 (29.8)	
Native American	33 (0.8)	NA^b^	48 (2.0)	
White	2279 (56.2)	390 (49.6)	1197 (50.8)	
Other	199 (4.9)	61 (7.8)	134 (5.7)	
Payer				
Medicare	25 (0.6)	NA^b^	15 (0.6)	.09
Medicaid	1982 (44.3)	414 (43.4)	1214 (46.0)	
Private	2022 (45.2)	459 (48.1)	1193 (45.2)	
Self-pay	278 (6.2)	49 (5.1)	160 (6.1)	
No charge	18 (0.4)	NA^b^	NA^b^	
Other	153 (3.4)	28 (2.9)	50 (1.9)	
Income, quartile				
First	1337 (30.3)	263 (28.2)	861 (33.5)	<.001
Second	1109 (25.1)	234 (25.1)	661 (25.7)	
Third	1033 (23.4)	211 (22.6)	543 (21.1)	
Fourth	933 (21.1)	226 (24.2)	503 (19.6)	

Abbreviations: CCI, Charlson Comorbidity Index; NA, not applicable; NOM, nonoperative management.

^a^ Total number may not be 8087 owing to weighted values. Data for most variables were missing in less than 1% of patients (0 patients for age, 0 patients for CCI score, 38 patients [0.5%] for location, 0 patients for region, 27 patients [0.3%] for payer, and 173 patients [2.1%] for income). Race and ethnicity data were missing in 891 patients (11.0%).

^b^ Suppressed per the Healthcare Cost and Utilization Project requirement.

^c^ Race and ethnicity categories reflect options provided for Healthcare Cost and Utilization Project coding of data elements.

Patients undergoing immediate operation were more likely to be White compared with patients who were successfully treated nonoperatively and those who failed nonoperative treatment and underwent delayed surgery. Similarly, Black patients represented a lower proportion of patients who underwent immediate operation compared with patients who trialed nonoperative treatment. There were 292 Black individuals (7.2%), 1095 Hispanic individuals (27.0%), and 2279 White individuals (56.2%) receiving immediate operation; 131 Black individuals (16.7%), 189 Hispanic individuals (24.0%), and 390 White individuals (49.6%) receiving successful nonoperative treatment; and 221 Black individuals (9.4%), 702 Hispanic individuals (29.8%), and 1197 White individuals (50.8%) receiving failed nonoperative treatment (*P* < .001). A higher proportion of successful nonoperative management of acute appendicitis occurred in urban teaching hospitals (594 patients [62.2%] vs 2418 patients [53.8%] receiving immediate operation and 1353 patients [51.1%] receiving failed nonoperative treatment in urban teaching hospitals), whereas a higher proportion of failed nonoperative management of acute appendicitis occurred in urban nonteaching hospitals (1078 patients [40.7%] vs 1685 patients [37.5%] receiving immediate operation and 307 patients [32.1%] receiving successful nonoperative treatment in urban nonteaching hospitals) (*P* < .001). Regional variabilities existed in patient presentation, with a higher proportion of patients coming from the South (eg, among patients receiving immediate operation, 1757 patients [39.1%] vs 639 patients [14.2%] from the Northeast, 908 patients [20.2%] from the Midwest, and 1188 patients [26.4%] from the West; *P* < .001). Additionally, patients who presented with complicated appendicitis were more likely to be in the first quartile of income (eg, among patients receiving immediate operation, 1337 patients [30.3%] vs 1109 patients [25.1%] in the second quartile, 1033 patients [23.4%] in the third quartile, and 933 patients [21.1%] in the fourth quartile; *P* < .001). No association between payer type and management strategy was identified (Table 2).

In multivariate logistic regression analysis, odds of preterm delivery, preterm labor, or abortion and odds of antepartum hemorrhage were not significantly different between immediate operation and successful nonoperative management. However, successful nonoperative management was associated with higher odds of premature rupture of membranes (OR, 2.77; 95% CI, 1.08-7.11; *P* = .03) compared with immediate operation (Table 3). Successful nonoperative management was also associated with higher odds of maternal infectious complications, including amniotic infection (OR, 4.35; 95% CI, 2.22-8.53; *P* < .001), pneumonia (OR, 2.52; 95% CI, 1.52-4.19; *P* < .001), and sepsis (OR, 1.52; 95% CI, 1.10-2.11; *P* = .01) compared with immediate operation. When a trial of nonoperative management was unsuccessful and followed by delayed operation, multivariate logistic regression found higher odds of preterm delivery, preterm labor, or abortion (OR, 1.45; 95% CI, 1.24-1.68; *P* < .001), as well as antepartum hemorrhage (OR, 1.56; 95% CI, 1.05-2.31; *P* = .03) and premature rupture of membranes (OR, 3.44; 95% CI, 1.56-7.61; *P* = .002) compared with immediate operation. In addition, compared with immediate surgery, failed nonoperative management with delayed operation was associated with higher odds of amniotic infection (OR, 4.74; 95% CI, 2.76-8.13; *P* < .001), pneumonia (OR, 2.01; 95% CI, 1.37-2.94; *P* < .001), and sepsis (OR, 1.58; 95% CI, 1.25-1.99; *P* < .001) (Table 3).

**Table 3.** Multivariate Logistic Regression for Maternal and Perinatal Complications

Outcome^a^	Successful NOM		Failed NOM	
	OR (95% CI)	*P* value	OR (95% CI)	*P* value
Preterm delivery, preterm labor, or abortion	1.08 (0.84-1.38)	.55	1.45 (1.24-1.68)	<.001
Antepartum hemorrhage	1.54 (0.89-2.66)	.12	1.56 (1.05-2.31)	.03
Premature rupture of membranes	2.77 (1.08-7.11)	.03	3.44 (1.56-7.61)	.002
Amniotic infection	4.35 (2.22-8.53)	<.001	4.74 (2.76-8.13)	<.001
Sepsis	1.52 (1.10-2.11)	.01	1.58 (1.25-1.99)	<.001
Pneumonia	2.52 (1.52-4.19)	<.001	2.01 (1.37-2.94)	<.001

Abbreviations: NOM, nonoperative management; OR, odds ratio.

^a^ Reference group for all comparisons is patients with immediate operations.

Median (IQR) LOS was 4 (3-6) days for immediate operation, 6 (3-6) days for successful nonoperative management, and 6 (4-9) days for failed nonoperative management. Compared with immediate operation, successful nonoperative management (regression coefficient [RC], 0.16; 95% CI, 0.14-0.18; *P* < .001) and failed nonoperative management (RC, 0.17; 95% CI, 0.15-0.19; *P* < .001) were associated with longer LOS. In addition, median (IQR) total hospital charges were $32 000 ($23 000-$49 000) for immediate operation, $38 000 ($20 000-$65 000) for successful nonoperative management, and $42 000 (IQR $27 000-$63 000) for failed nonoperative management. Successful (RC, 0.09; 95% CI, 0.07-0.11; *P* < .001) and unsuccessful (RC, 0.12; 95% CI, 0.11-0.14; *P* < .001) nonoperative management were also associated with increased hospital charges compared with immediate operation.

In a subgroup analysis of patients undergoing an operation and examining the association of delay in operative intervention with maternal and perinatal outcomes, multivariate logistic regression analysis found that delays to surgery were uniformly associated with higher odds of complications (Table 4). Each day in delay to surgery was associated with an increase in odds of preterm delivery, preterm labor, or abortion by 23% (OR, 1.23; 95% CI, 1.18-1.29; *P* < .001). Antepartum hemorrhage (OR, 1.29; 95% CI, 1.16-1.42; *P* < .001) and premature rupture of membranes (OR, 1.45; 95% CI, 1.26-1.67; *P* < .001) also had higher odds per day in delay. Each day in delay to operation was also associated with higher odds of maternal infectious complications, including amniotic infection (OR, 1.38; 95% CI, 1.24-1.53; *P* < .001), sepsis (OR, 1.13; 95% CI, 1.06-1.22; *P* = .001), and pneumonia (OR, 1.15; 95% CI, 1.04-1.27; *P* = .005) (Table 4).

**Table 4.** Multivariate Logistic Regression for Association Between Delay in Operation and Complications^a^

Outcome	OR (95% CI) per d of delay	*P* value
Preterm delivery, preterm labor, or abortion	1.23 (1.18-1.29)	<.001
Antepartum hemorrhage	1.29 (1.16-1.42)	<.001
Premature rupture of membranes	1.45 (1.26-1.67)	<.001
Amniotic infection	1.38 (1.24-1.53)	<.001
Sepsis	1.13 (1.06-1.22)	.001
Pneumonia	1.15 (1.04-1.27)	.005

Abbreviation: OR, odds ratio.

^a^ Subgroup analysis was performed only among patients undergoing operative intervention.

## Discussion

This cohort study found that 56% of pregnant women with complicated appendicitis underwent an immediate operative intervention. This is lower than the recently published 85% appendectomy rate for all pregnant women with appendicitis, combining uncomplicated and complicated appendicitis from 28 hospitals in the US.^26^ This is likely associated with well-defined recommendations supporting immediate surgery for uncomplicated appendicitis; however, no definitive guidelines have been established on how best to manage complicated appendicitis among pregnant patients. Therefore, it is critical to understand the association of operative and nonoperative management of acute appendicitis in this special population with maternal and perinatal outcomes. To our knowledge, this is the largest study using a nationwide database to find significant benefits in outcomes associated with immediate operation during pregnancy in patients with complicated appendicitis.

We found that immediate operation was associated with lower odds of maternal infectious complications, including amniotic infection, pneumonia, and sepsis, compared with successful and unsuccessful nonoperative management, with no association with odds of preterm delivery, preterm labor, or abortion or with antepartum hemorrhage. It is also important to note that a trial of nonoperative management is not always successful. In this study, 74% of pregnant women who trialed nonoperative management of complicated appendicitis failed and subsequently underwent operative intervention during the same hospital stay. In this group of women, maternal and perinatal outcomes were uniformly worse compared with the immediate operation group. Furthermore, each day in delay to surgery was associated with higher odds of complication for every maternal and perinatal outcome assessed in this study. Additionally, as hospital cost containment and LOS play a greater role in decision-making, it is important to note that immediate operation was associated with shorter hospital stays and decreased hospital costs.

Existing literature that evaluates appendectomy during pregnancy tends to focus on uncomplicated appendicitis or does not define severity of presentation.^2,4,8,9,27^ National guidelines like those published by the Society of American Gastrointestinal and Endoscopic Surgeons strongly support appendectomy in acute uncomplicated appendicitis in pregnant women, going so far as to state, “There is no role for nonoperative management of uncomplicated acute appendicitis.”^11^ In contrast, sparse guidance exists for the management of complicated appendicitis during pregnancy. Pregnant women with complicated appendicitis are unlike pregnant women with uncomplicated appendicitis given that complications like perforation are clearly associated with increased maternal and fetal morbidity; however, it is unclear how these elevated stakes should affect treatment decisions.^12^ Studies^15,28^ in the general population have found that complicated appendicitis is also associated with increased morbidity compared with uncomplicated appendicitis, with management historically focused on antibiotics and percutaneous drainage, with or without interval appendectomy. However, updated consensus guidelines for the management of complicated appendicitis in the general population have suggested the feasibility and safety of laparoscopic appendectomy, with an association with shorter hospital stays and decreased morbidity and mortality compared with nonoperative intervention.^6,15,17,18^ In their retrospective cohort study in a general population presenting with complicated appendicitis, excluding pregnant patients, Nimmagadda et al^29^ found a similar difference in outcomes between immediate operation and successful nonoperative intervention as in our study, with immediate operation associated with a shortened hospital LOS. However, in their population, the failure rate for nonoperative management was 13.9% compared with the greater than 70% failure rate observed in our study, with both studies finding increased morbidity and LOS in those patients who failed a trial of nonoperative management of acute appendicitis. These results suggest that our study findings may help define the preferred management strategy in complicated appendicitis during pregnancy to be immediate operation.

### Limitations

There are several limitations to this study. The NIS is a retrospective administrative database that includes patients based on discharge diagnoses, leading to possible misclassification bias as the diagnoses evaluated in this study would be subject to facility-level definitions. Similarly, the NIS does not maintain longitudinal follow-up data for its patients; therefore, it fails to capture complications manifesting on readmission encounters, which can yield unmeasured confounding. Given that successful nonoperative management in this study was defined as not having undergone an operation during the hospital stay being examined, the number of readmissions for these patients was not quantified. Previous literature suggests that the recurrence rate after nonoperative management of appendicitis and subsequent operation can be as high as 30%.^30^ Our study was unable to include clinical and economic outcomes associated with these recurrences. Additionally, this study was unable to capture clinical data to delineate why patients may have failed nonoperative treatment. Prior published data suggests reasons for nonoperative failure may include persistent pain and signs of systemic infection, including tachycardia and fever.^29^ Additional considerations must be made for the fetus in pregnant patients, and therefore a prospective study to understand nonoperative failure is warranted. Interestingly, among patients in our study who failed nonoperative treatment and required operative intervention, an increased proportion underwent open appendectomy compared with laparoscopy. There may be many reasons for a surgeon to choose an open surgical approach over laparoscopy that we were unable to define in this study; this warrants further investigation. Another limitation of this study is the inability of *ICD-9-CM* codes to reflect trimesters in pregnancy. Operative and nonoperative risks are likely to differ across trimesters, although current literature suggests that operative intervention is safe in any trimester.^7,27,31^ Additionally, our study does not capture the outcomes associated with negative appendectomy during pregnancy, which has been associated with increased maternal morbidity, preterm labor, and fetal loss.^10,32^ However, our data used discharge diagnosis codes and so was unlikely to include patients with a negative appendectomy given that the diagnosis was eventually confirmed prior to discharge.

## Conclusions

These results suggest that immediate operative intervention may be considered as the first-line treatment for pregnant women presenting with complicated appendicitis. Our findings suggest that, at best, even if nonoperative management is successful, it would not be associated with superior outcomes compared with immediate operation. Furthermore, nonoperative management frequently fails, leading to delayed operative intervention, which is associated with worse clinical outcomes. Additional research is required to examine the longitudinal, long-term outcomes associated with operative and nonoperative management of complicated appendicitis in this unique patient population.

## References

[1] Abbasi N, Patenaude V, Abenhaim HA. Evaluation of obstetrical and fetal outcomes in pregnancies complicated by acute appendicitis. Arch Gynecol Obstet. 2014;290(4):661-667. doi:10.1007/s00404-014-3276-7 24838290

[2] Andersen B, Nielsen TF. Appendicitis in pregnancy: diagnosis, management and complications. Acta Obstet Gynecol Scand. 1999;78(9):758-762. doi:10.1034/j.1600-0412.1999.780903.x 10535336

[3] Mourad J, Elliott JP, Erickson L, Lisboa L. Appendicitis in pregnancy: new information that contradicts long-held clinical beliefs. Am J Obstet Gynecol. 2000;182(5):1027-1029. doi:10.1067/mob.2000.105396 10819817

[4] Won RP, Friedlander S, Lee SL. Management and outcomes of appendectomy during pregnancy. Am Surg. 2017;83(10):1103-1107. doi:10.1177/000313481708301018 29391104

[5] Cox TC, Huntington CR, Blair LJ, . Laparoscopic appendectomy and cholecystectomy versus open: a study in 1999 pregnant patients. Surg Endosc. 2016;30(2):593-602. doi:10.1007/s00464-015-4244-4 26091987

[6] Korndorffer JR, Fellinger E, Reed W. SAGES guideline for laparoscopic appendectomy. Surg Endosc. 2010;24(4):757-761. doi:10.1007/s00464-009-0632-y 19787402

[7] Rollins MD, Chan KJ, Price RR. Laparoscopy for appendicitis and cholelithiasis during pregnancy: a new standard of care. Surg Endosc. 2004;18(2):237-241. doi:10.1007/s00464-003-8811-8 14691706

[8] Abbasi N, Patenaude V, Abenhaim HA. Management and outcomes of acute appendicitis in pregnancy-population-based study of over 7000 cases. BJOG. 2014;121(12):1509-1514. doi:10.1111/1471-0528.12736 24674238

[9] Silvestri MT, Pettker CM, Brousseau EC, Dick MA, Ciarleglio MM, Erekson EA. Morbidity of appendectomy and cholecystectomy in pregnant and nonpregnant women. Obstet Gynecol. 2011;118(6):1261-1270. doi:10.1097/AOG.0b013e318234d7bc 22105255 PMC3702040

[10] Cheng HT, Wang YC, Lo HC, . Laparoscopic appendectomy versus open appendectomy in pregnancy: a population-based analysis of maternal outcome. Surg Endosc. 2015;29(6):1394-1399. doi:10.1007/s00464-014-3810-5 25171885

[11] Pearl JP, Price RR, Tonkin AE, Richardson WS, Stefanidis D. SAGES guidelines for the use of laparoscopy during pregnancy. Surg Endosc. 2017;31(10):3767-3782. doi:10.1007/s00464-017-5637-3 28643072

[12] Orbay H, Kariya CM, Kavic SM. Acute appendicitis during pregnancy. In: Nezhat CH, Kavic MS, Lanzafame RJ, Lindsay MK, Polk TM, eds. Non-Obstetric Surgery During Pregnancy: A Comprehensive Guide. Springer International Publishing; 2019:135-146.

[13] Segev L, Segev Y, Rayman S, Nissan A, Sadot E. Acute appendicitis during pregnancy: different from the nonpregnant state? World J Surg. 2017;41(1):75-81. doi:10.1007/s00268-016-3731-7 27730353

[14] Bahram MA. Evaluation of early surgical management of complicated appendicitis by appendicular mass. Int J Surg. 2011;9(1):101-103. doi:10.1016/j.ijsu.2010.10.006 20965290

[15] Mentula P, Sammalkorpi H, Leppäniemi A. Laparoscopic surgery or conservative treatment for appendiceal abscess in adults: a randomized controlled trial. Ann Surg. 2015;262(2):237-242. doi:10.1097/SLA.0000000000001200 25775072

[16] Young KA, Neuhaus NM, Fluck M, . Outcomes of complicated appendicitis: is conservative management as smooth as it seems? Am J Surg. 2018;215(4):586-592. doi:10.1016/j.amjsurg.2017.10.032 29100591

[17] Guller U, Jain N, Peterson ED, Muhlbaier LH, Eubanks S, Pietrobon R. Laparoscopic appendectomy in the elderly. Surgery. 2004;135(5):479-488. doi:10.1016/j.surg.2003.12.007 15118584

[18] Kouwenhoven EA, Repelaer van Driel OJ, van Erp WF. Fear for the intraabdominal abscess after laparoscopic appendectomy: not realistic. Surg Endosc. 2005;19(7):923-926. doi:10.1007/s00464-004-2083-9 15920693

[19] Healthcare Cost and Utilization Project. Overview of the National (Nationwide) Inpatient Sample. Agency for Healthcare Research and Quality. Accessed March 2, 2020. https://www.hcup-us.ahrq.gov/nisoverview.jsp

[20] Tashiro J, Einstein SA, Perez EA, Bronson SN, Lasko DS, Sola JE. Hospital preference of laparoscopic versus open appendectomy: effects on outcomes in simple and complicated appendicitis. J Pediatr Surg. 2016;51(5):804-809. doi:10.1016/j.jpedsurg.2016.02.028 26944182

[21] Saluja S, Sun T, Mao J, . Early versus late surgical management of complicated appendicitis in children: a statewide database analysis with one-year follow-up. J Pediatr Surg. 2018;53(7):1339-1344. doi:10.1016/j.jpedsurg.2017.09.012 29032983

[22] Healthcare Cost and Utilization Project. NEOMAT—neonatal and/or maternal *ICD-9-CM* DX and/or PR. Agency for Healthcare Research and Quality. Accessed March 2, 2020. https://www.hcup-us.ahrq.gov/db/vars/neomat/nisnote.jsp

[23] Quan H, Sundararajan V, Halfon P, . Coding algorithms for defining comorbidities in *ICD-9-CM* and *ICD-10* administrative data. Med Care. 2005;43(11):1130-1139. doi:10.1097/01.mlr.0000182534.19832.83 16224307

[24] Bureau of Labor Statistics. Consumer Price Index inflation calculator. Accessed December 10, 2020. https://data.bls.gov/cgi-bin/cpicalc.pl

[25] Henry AJ, Hevelone ND, Lipsitz S, Nguyen LL. Comparative methods for handling missing data in large databases. J Vasc Surg. 2013;58(5):1353-1359.e6. doi:10.1016/j.jvs.2013.05.008 23830314

[26] Vasileiou G, Eid AI, Qian S, ; EAST Appendicitis Study Group. Appendicitis in pregnancy: a post-hoc analysis of an EAST Multicenter Study. Surg Infect (Larchmt). 2020;21(3):205-211. doi:10.1089/sur.2019.102 31687887

[27] Affleck DG, Handrahan DL, Egger MJ, Price RR. The laparoscopic management of appendicitis and cholelithiasis during pregnancy. Am J Surg. 1999;178(6):523-529. doi:10.1016/S0002-9610(99)00244-5 10670865

[28] Mällinen J, Rautio T, Grönroos J, . Risk of appendiceal neoplasm in periappendicular abscess in patients treated with interval appendectomy vs follow-up with magnetic resonance imaging: 1-year outcomes of the Peri-Appendicitis Acuta randomized clinical trial. JAMA Surg. 2019;154(3):200-207. doi:10.1001/jamasurg.2018.4373 30484824 PMC6439633

[29] Nimmagadda N, Matsushima K, Piccinini A, . Complicated appendicitis: immediate operation or trial of nonoperative management? Am J Surg. 2019;217(4):713-717. doi:10.1016/j.amjsurg.2018.12.061 30635209

[30] Salminen P, Tuominen R, Paajanen H, . Five-year follow-up of antibiotic therapy for uncomplicated acute appendicitis in the APPAC randomized clinical trial. JAMA. 2018;320(12):1259-1265. doi:10.1001/jama.2018.13201 30264120 PMC6233612

[31] Stepp K, Falcone T. Laparoscopy in the second trimester of pregnancy. Obstet Gynecol Clin North Am. 2004;31(3):485-496, vii. doi:10.1016/j.ogc.2004.05.002 15450312

[32] Aggenbach L, Zeeman GG, Cantineau AE, Gordijn SJ, Hofker HS. Impact of appendicitis during pregnancy: no delay in accurate diagnosis and treatment. Int J Surg. 2015;15:84-89. doi:10.1016/j.ijsu.2015.01.025 25638737

